# Supplementary material for: Identification of photoperiod-induced specific miRNAs in the adrenal glands of Sunite sheep (Ovis aries)
Source: Front Vet Sci. 2022 Jul 22;9:888207. doi: 10.3389/fvets.2022.888207 (PMC9354845; doi:10.3389/fvets.2022.888207)
Supplement: Supplementary file 1 [file Data_Sheet_1.ZIP › Supplementary file/miRDeep2 file.zip!/miRDeep2 file/Know_Structure/LP42A1/oar-mir-22.pdf]

miRBase precursor : oar-mir-22
Total read count : 126899
oar-miR-22-3p read count : 120190
remaining reads : 6709

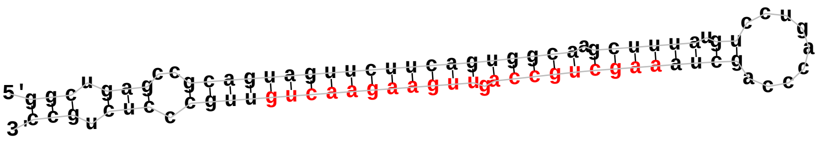

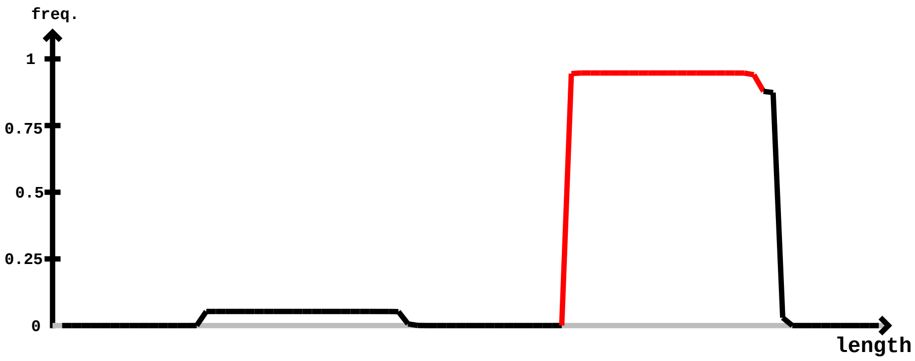

oar-miR-22-3p

5'- ggcugagccgcaguaguucuucaguggcaagcuuuauguccugacccagcuaaagcugccaguugaagaacuguugcccucugcc -3'	exp		
(((.(((..((((((((((((((((((((.((((((.((.........))))))))))))).))))))))))))))).))).)))	reads	mm	sample
.............uaguucuucaguggcaagcuuu..	12	0	seq
..............aguucuucaguggcaagc...	4	0	seq
..............aguucuucaguggcaagcu..	10	0	seq
..............aguucuucaguggcaagcuu...	67	0	seq
..............aguucuucaguggcaagcuuu..	5807	0	seq
..............aguucuucaguggcaagcuuua...	742	0	seq
..............aguucuucaguggcaagcuuuau..	58	0	seq
...............guucuucaguggcaagcuuu..	7	0	seq
...............guucuucaguggcaagcuuua...	1	0	seq
................................uuuauguccugacccagcuaaagcugcc.........................	1	0	seq
....aaagcugccaguugaagaacu.............	4	0	seq
....aaagcugccaguugaagaacugu...........	17	0	seq
....aaagcugccaguugaagaacuguu..........	1	0	seq
....aagcugccaguugaaga................	11	0	seq
....aagcugccaguugaagaa...............	26	0	seq
....aagcugccaguugaagaac..............	811	0	seq
....aagcugccaguuCaagaacu.............	1	1	seq
....aagcugccaguugaagaacu.............	7845	0	seq
....aagcugccaguugaagaacug............	594	0	seq
....aagcugccaguugaagaacugu...........	106856	0	seq
....aagcugccaguugaagaacuguu..........	3710	0	seq
....aagcugccaguugaagaacuguug.........	3	0	seq
....agcugccaguugaagaacu.............	32	0	seq
....agcugccaguugaagaacug............	2	0	seq
....agcugccaguugaagaacugu...........	246	0	seq
....agcugccaguugaagaacuguu..........	27	0	seq
....gcugccaguugaagaacugu...........	2	0	seq
....cugccaguugaagaacugu...........	1	0	seq
....ugccaguugaagaacugu...........	1	0	seq
